# Mathematical modelling of exponential growth as a rich learning environment for mathematics classrooms

**DOI:** 10.1007/s11858-022-01433-8

**Published:** 2022-10-10

**Authors:** Hans-Stefan Siller, Hans-Jürgen Elschenbroich, Gilbert Greefrath, Katrin Vorhölter

**Affiliations:** 1 University of Wuerzburg, Wuerzburg, Germany; 2 Medienberatung NRW, Düsseldorf, Germany; 3 University of Muenster, Muenster, Germany; 4 University of Hamburg, Hamburg, Germany

## Abstract

Mathematical concepts are regularly used in media reports concerning the Covid-19 pandemic. These include growth models, which attempt to explain or predict the effectiveness of interventions and developments, as well as the reproductive factor. Our contribution has the aim of showing that basic mental models about exponential growth are important for understanding media reports of Covid-19. Furthermore, we highlight how the coronavirus pandemic can be used as a context in mathematics classrooms to help students understand that they can and should question media reports on their own, using their mathematical knowledge. Therefore, we first present the role of mathematical modelling in achieving these goals in general. The same relevance applies to the necessary basic mental models of exponential growth. Following this description, based on three topics, namely, investigating the type of growth, questioning given course models, and determining exponential factors at different times, we show how the presented theoretical aspects manifest themselves in teaching examples when students are given the task of reflecting critically on existing media reports. Finally, the value of the three topics regarding the intended goals is discussed and conclusions concerning the possibilities and limits of their use in schools are drawn.

## Introduction

The exponential growth of infections is considered by scientists to be among the most important indicators for describing and evaluating their spread, and especially for explaining the spread of Sars-Cov-2, which causes the widely known Covid-19 disease. The media have picked up on the topic of exponential growth in their headlines and articles, in turn placing mathematics front and centre in society. However, it is not only through the media that references have been made using mathematical concepts; politicians around the world are also frequently using terms such as reproduction value, growth rate, growth factor and doubling time in their arguments for or explanations of measures taken during the pandemic.

Thus, due to the pandemic, the mathematical concept of exponential growth has achieved considerable social prominence, although for many people, this awareness is not necessarily accompanied by an understanding of it. As Hutzler et al., (2021) put it, “Humans are woefully inept at intuitively grasping exponential growth functions. Lay persons as well as political decision makers grossly underestimate exponential growth […], regardless of being presented with numerical, graphical or non-quantitative representations […]” (p. 1).

An important goal in mathematics education is to teach mathematical concepts that are relevant in the real world in a way that centres on promoting understanding of them. It is therefore obvious that mathematical modelling should have a significant contribution to sense-making in mathematics education (Stillman et al., 2020). In order to enable mathematical methods to be applied to real-life problems on exponential growth, such as in the case of the Covid-19 pandemic, so-called basic mental models (*Grundvorstellungen*) of mathematical content are necessary (Hefendehl-Hebeker et al., 2019). These basic mental models form an indispensable bridge between a mathematical model and the reality-based situation to be described (Kleine et al., 2005, p. 229).

Modelling activities require a conceptual understanding of the mathematics in question (Blomhøj, 2004). The teaching of mathematical content by means of “applications and modelling play an important role in teaching and learning of mathematics; already in the nineteenth century, famous mathematics educators made a strong plea for the inclusion of contextual problems in mathematics education” (Kaiser, 2020, p. 554).

Against the background of the Covid-19 pandemic, our contribution shows that the concept of exponential growth and its limits should be part of everyone’s general education (Bakker et al., 2021). As stated above, the concept of exponential growth is still insufficiently developed today. Many people do not have a sense of exponential processes and, likewise, of their limits (Lammers et al., 2020). Thus, we highlight not only the importance but also the limitations of the exponential growth model as curriculum content. Our approach establishes a connection of mathematical modelling from the perspective of basic mental models, and it is directly realisable in curricula. In this way, we provide an example to demonstrate—based on the Covid-19 pandemic—which possibilities for understanding the rest of the world (Blum & Leiß, 2007, p. 225) can be realised in the classroom, so that students become responsible citizens (Kaiser, 2020). This can be done by using basic mental models for understanding, such as reports of Covid-19 in the media. Our considerations and resulting recommendations are based on the importance of understanding exponential growth in the context of the Covid-19 pandemic, but also seem relevant to future curricular decisions, perhaps resonating with the work of of Engelbrecht et al., (2020).

## Mathematical Modelling

Mathematical modelling involves developing a simplified mathematical description of real-life problems, working within a mathematical model and then interpreting and validating the mathematical results thus obtained in relation to the real-life problem (Niss et al., 2007). Thus, the concept of modelling in mathematics classrooms refers to the “process of solving real-world problems by means of mathematics” (Blum, 2015, p. 77) and in particular the application of mathematics in real and meaningful contexts using real-world problems, issues or contexts in an educational context.

A mathematical model can therefore be conceived as a mapping from reality to the mathematical world (Niss et al., 2007, p. 4), as it simplifies only certain sufficiently objectifiable partial aspects of the rest of the world. Reality or real facts are deliberately simplified by mathematical modelling, and the exactness with which they can be represented is always limited. Models can be normative and descriptive and therefore serve different functions (Greefrath & Vorhölter, 2016).

**Fig. 1:**
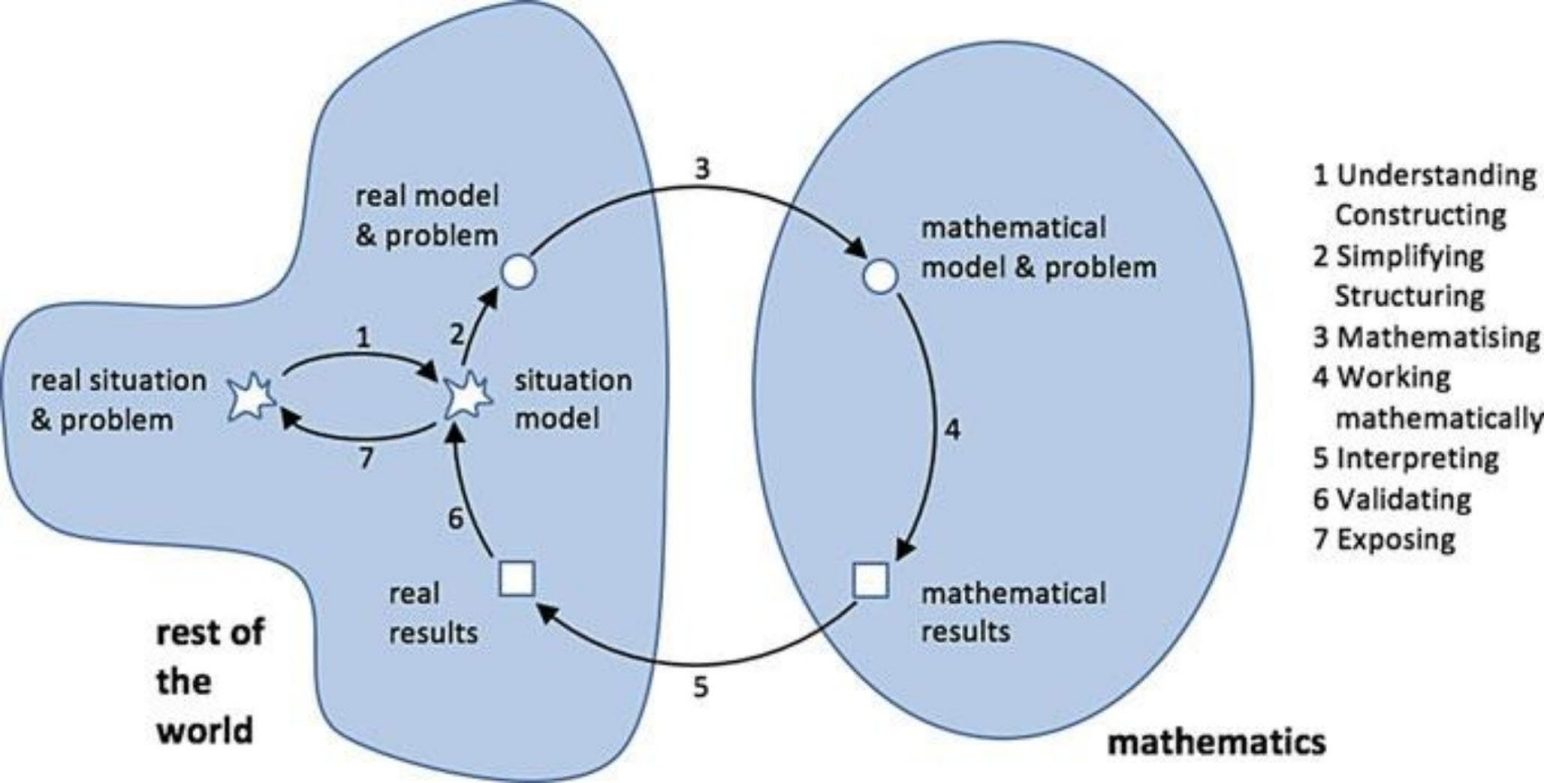
Modelling cycle according to Blum & Leiß (2007, p. 225)

Running through the various sub-steps multiple times can be idealised as a modelling cycle, such as the one illustrated by Blum & Leiß (2007, p. 225) in Fig. 1. The processing of a problem in order to transfer phenomena of the extra-mathematical world into the world of mathematics in a simplified way and, after working on a mathematical model, to return the mathematical results to the extra-mathematical world (Niss et al., 2007) requires modelling competence, which expresses itself by working “autonomously and insightfully […] through all aspects of a mathematical modelling process in a certain context” (Blomhøj & Jensen, 2003, p. 126).

The ability to carry out a sub-process in the modelling cycle can be regarded as a specific sub-competence of mathematical modelling—in contrast to modelling competence, which refers to the ability to carry out an entire modelling process and reflect on it. The possession of these sub-competencies may be regarded as a necessary prerequisite for the successful completion of a modelling process. These are not sufficient, but must be supplemented by global modelling competencies (Kaiser, 2007; Maaß, 2006). In the international literature, this discussion has been conducted under the label of “holistic vs. atomistic approach”, according to Blomhøj & Jensen (2003). In this paper, we concentrate on the holistic approach. This is justified by a study by Kaiser & Brand (2015), which showed that the promotion of modelling competencies is most effective when whole modelling tasks have to be worked on. A holistic approach succeeds in promoting “overall goals”, which include students being able to perform “applicational/modelling/applied problem solving processes” to “acquire knowledge of existing models”, as well as being “able to critically analyse and assess given examples of modelling and applications” (Blum & Niss, 1991, p. 44–45). Furthermore, corresponding to the “mixing approach” for teaching mathematical modelling,

elements of applications and modelling are invoked to assist the introduction of mathematical concepts etc. Conversely, newly developed mathematical concepts, methods and results are activated towards applicational and modelling situations whenever possible. In this approach, the mathematics to be involved in applications and modelling activities is more or less given from the outset. (Blum & Niss, 1991, p. 61)

The development of mathematical models thus makes it possible to actively pursue mathematics in real-life contexts (Siller & Greefrath, 2010). In the context of real problems, mathematically important and relevant aspects can be identified, realised, implemented and tested. In addition, the context of the modelling problem itself, or indeed the act of working through it, can provide students with an offer of meaning that they can (but do not have to) accept. This can lead to their experiencing mathematical content, or even participating in mathematics, in a way that is meaningful to them (Vorhölter & Schwarz, 2020).

Nevertheless, students should always be aware that reality is more complex than mathematical models, and even more complex than the simplified models that are considered at school. Nevertheless, even simple models present the opportunity to vary their parameters and to investigate the effects and interactions of doing so.

## Teaching exponential growth

Especially at times of national and international challenges, such as the handling of the Covid-19 pandemic, demands for the use of daily published figures of infected persons, for example, are often voiced for elementary mathematical consideration. To understand and comprehend the spread of the coronavirus and to be able to question the meaningfulness of measures taken and restrictions imposed, the concept of exponential growth is essential.

### Importance of exponential growth for understanding pandemics

The concept of exponential growth challenges arose due to the so-called exponential growth bias (Levy & Tasoff, 2017), which had already been reported long before the pandemic in connection with the misperception of exponential growth (Keren, 1983; Wagenaar & Sagaria, 1975). The crucial issue regarding this exponential growth bias “is the phenomenon that humans intuitively under-estimate exponential growth” (Schonger & Sele, 2021, p. 221).

There is little understanding of exponential growth among large parts of the population. This has been shown time and again by astonished reactions to tasks from popular mathematics, such as the following:

- How many grains of wheat will fill a chess board if the number continues to double from square to square?
- How many days does it take for a pond to be half-covered by fast-growing algae that doubles in size every day, if the whole pond is completely covered after 60 days?

On the one hand, the audience is usually impressed by such examples, but on the other, they are left with a strange ‘feeling of not understanding’. The legend of the grains of wheat on the chessboard cannot be real if the last square is equivalent to the global harvest of several centuries. And if colonization with algae or a viral infection would really progress permanently and exponentially, the whole planet would be overwhelmed in a short time, or all mankind would be infected. The exponential growth model can therefore be used only for meaningful intervals. Nevertheless, an adequate conception of exponential growth can be built up only gradually through a systematic study of exponential growth and decay processes based on sensibly chosen examples and contexts.

Knowledge and understanding of exponential growth, in particular with regard to knowledge about other growth processes such as linear or logistic growth, should be part of mathematics education. Too much focus on linear processes alone can lead to misconceptions (Bock et al., 2002). This does not require particularly complex mathematical content, such as differential equations; a basic understanding can even be acquired at secondary school level.

In relation to the Covid-19 pandemic, difficulties in the acceptance of *social distancing* due to the lack of conceptions about exponential growth as reported by Lammers et al., (2020) should be mentioned, who emphasised that “overcoming this bias increases support for social distancing” (Lammers et al., 2020, p. 16,264). In this context, the authors of the study stressed that “the coronavirus outbreak is a unique moment in history that directly impacts people’s deepest concerns about their lives and those of their loved ones” (Lammers et al., 2020, p. 16,265).

### Basic mental models (*Grundvorstellungen*) for exponential growth

The concept of basic mental models has been well established in German-speaking mathematics education for many years. It is used to describe the content-related meaning that learners should attribute to mathematical concepts (Vom Hofe & Blum, 2016).

In mathematics, exponential growth is understood to be a process in which a stock quantity multiplies by the same percentage in equal units. This describes the characteristic property for this process, which contains the core of the meaning. The concept formation process in dealing with exponential growth in learning and related difficulties has been addressed in several studies, including those by Castillo-Garsow (2013), Confrey & Smith (1994) and Ellis et al., (2015).

**Fig. 2:**
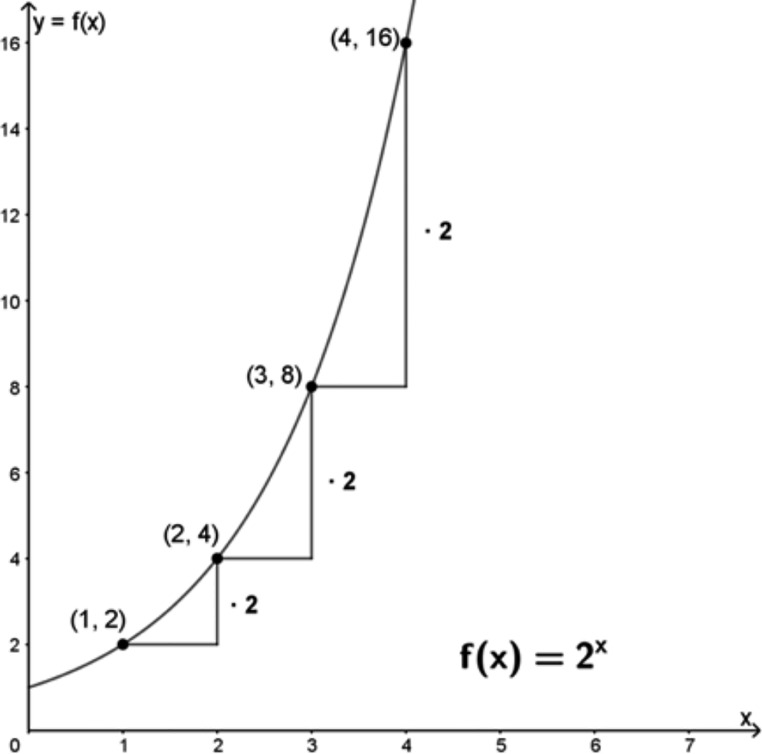
Constancy of the factor in exponential growth

From an algebraic perspective, exponential growth is described symbolically with the aid of an equation of the form of $f\left({x}_{1}+{x}_{2}\right)=f\left({x}_{1}\right)\bullet f\left({x}_{2}\right)$; that is, if the argument in exponential growth is increased by the same value in each case, the function value also increases by the same factor in those cases (cf. Figure 2).

While the increase of the argument is additive, the function value grows multiplicatively.

On this basis, essential ideas about exponential growth can also be described from an application-orientated perspective (Ärlebäck et al., 2013; Bush et al., 2015; Russo et al., 2020). Graphically, the crucial notion for exponential growth is that of the stock curve increasing more and more. We find such a trajectory, for example, at the onset of epidemics and pandemics. However, this also includes the awareness that this cannot occur indefinitely, but only for a limited period of time.

We focus here on basic mental models for exponential growth (Hußmann & Prediger, 2016), which are similarly based on different categorisations (Thiel-Schneider, 2018, p. 32 ff.), as follows:

1. Analytically, exponential growth means that growth is proportional to the current stock; that is, growth by the same factor occurs in the same unit.

2. Graphically, exponential growth means an ever-steeper rise in the stock curve

3. Symbolically, exponential growth associates ${N}_{new}={N}_{new-1}+Increment$ with $Increment \propto {N}_{new-1}$ recorded.
4. The growth factor is the ratio $\frac{\varDelta {N}_{new}}{\varDelta {N}_{new-1}}$.

These basic mental models focus on different aspects of exponential growth and represent it in the way described by Hutzler et al., (2021) at the beginning, i.e. numerically, graphically and non-quantitatively. It is important that students not only develop the abovementioned basic mental models for exponential growth per se but are also able to distinguish such growth from other types. They should not only be able to graphically distinguish prototypical representations of different growth processes from each other but should also know analytically, for example, the difference between linear and exponential growth as the same increase independent of the existing stock, on the one hand, and as proportional to the existing stock, on the other. Therefore, they should also be able to distinguish between the different growth factors.

## Examples for modelling with and through exponential growth based on basic mental models

Detached from all medical, ethical, social and natural issues, the Covid-19 pandemic can be understood as an opportunity to develop an understanding of exponential growth in terms of the basic mental models concerning it. In the following, we use three different topics to illustrate the extent to which the basic mental models help to understand the topics, and to show the extent to which the development of such models is promoted, and which sub-processes of mathematical modelling are addressed by working through the individual questions. Furthermore, dealing with these examples leads to the development of critical and responsible citizens. We follow a pragmatic approach, which should be understood as a complement to existing data-based approaches in the literature. For instance Heyd-Metzuyanim et al., (2021) used media coverage of Covid-19 as a starting point to examine the public’s understanding of mathematical concepts needed to understand the pandemic and to predict its spread. The results presented suggest that mathematics in school, taught at a higher level, can prepare adults to understand critical information important to their well-being, for example, in times of a global pandemic. However, it is also clear that a weak mathematical identity, used here to describe a wide range of non-cognitive relations to mathematics, significantly hinders the engagement of relevant information. We complement this approach with basic mental models about exponential growth. In this way, it can be possible to take up and implement authentic situations—such as the media coverage just described—as a starting point for mathematical modelling. Even though the approach of data-based modelling is seen as an important development, it must also be noted that this makes many demands on teachers (Lesh, 2010). Another linkage point for mathematical modelling may be that of Kwon et al., (2021), who uses visual representations in the news media during escalating events such as the Covid-19 pandemic as a starting point. In doing so, the authors examined the use of graphs in Korean news media during the outbreak of the Covid-19 pandemic, using a quantitative analysis to examine the type and frequency of graphics used in Covid-19 news stories and a qualitative analysis to examine the content of news stories with graphs. There were also links to mathematical modelling and the need for basic mental models, as a basic understanding of exponential growth seems to be necessary to implement adequate mathematical modelling; the basic mental models then help to construct a suitable mathematization or mathematical model. Our approach can therefore be understood as a consistent further development of this idea. This is underlined by Aguilar & Castaneda (2021). They investigated the influence of mathematical competence on health literacy and identified or characterised mathematical competences a citizen needs to interpret the official information about the Covid-19 pandemic. This process also requires citizens to have a certain level of modelling competence and basic mental models. Society must therefore be sensible to acquire statistical thinking in order to interpret the information disseminated daily by the media (Da Silva et al., 2021). This is all essential because the development of critical citizens can only be attained if a certain degree of questioning of content is possible, i.e., if basic mental models are explicitly realized in mathematical modelling. The contribution by Krause et al., (2021), questioning how school mathematics can prepare citizens for a democratic society, can act as a parenthesis. In particular, the question raised in this paper, “what is needed to navigate this complex situation that involves, among other things, mathematics?“ (p. 87), which is reflected by three narratives from three countries, can be used as a starting point for mathematical modelling and the use of basic mental models. There are connections to the contribution by Krause et al., as “the development of a positive attitude towards mathematics in order to deal with real-world problems and to reflect on them” is particularly emphasized.

In none of these contributions are mathematical modelling and necessary content-related prerequisites made as explicit as we do here. We focus on the aspects of a modelling process with functional mathematical models, namely making assumptions, mathematising, working mathematically, interpreting and validating. This analysis is also supported by the contribution of Geiger and Gal (2022), who provide insights into the multiple capabilities of citizens to engage with mass media demands, to critically analyse statistical and mathematical information in the media, to evaluate the meaning and credibility of reports, and to understand and make informed judgements about public policies.

In order to illustrate this pragmatic approach step by step, we first introduce different growth approaches (Sect. 4.1), model the course of infection under different assumptions (Sect. 4.2) and show how students can identify and realise corresponding confidence intervals (Fig. 7) and look at the growth factor at different points in time (Sect. 4.3).

### Investigation of the type of infection growth

**Fig. 3:**
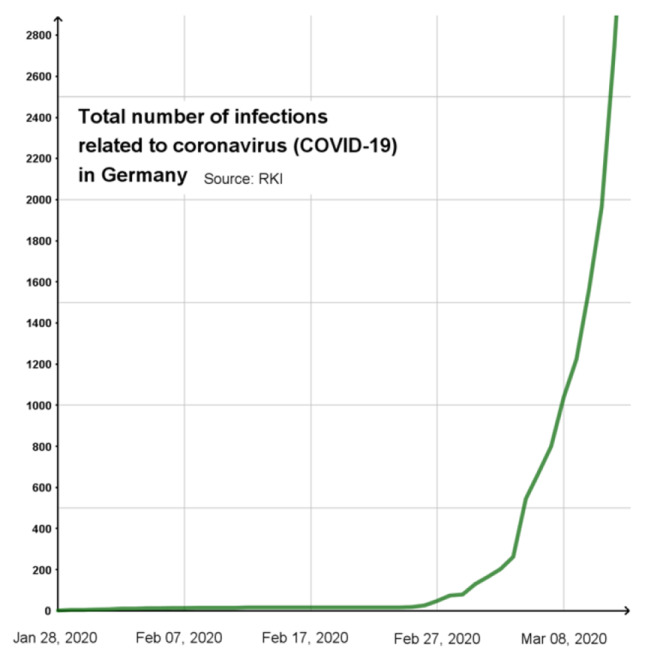
Total number of infections related to Covid-19 in Germany (January 2020–March 2020) showing exponential growth

The starting point for investigating the type of infection growth might be a graph in a daily newspaper; analogously, data can be prepared by a teacher (cf. Figure 3). The associated question could concern which type of function could best describe the growth. Using the terms from the discourse on mathematical modelling, this means finding a suitable mathematical model as a means to mathematise the real model (step 3 in Fig. 1).

**Fig. 4:**
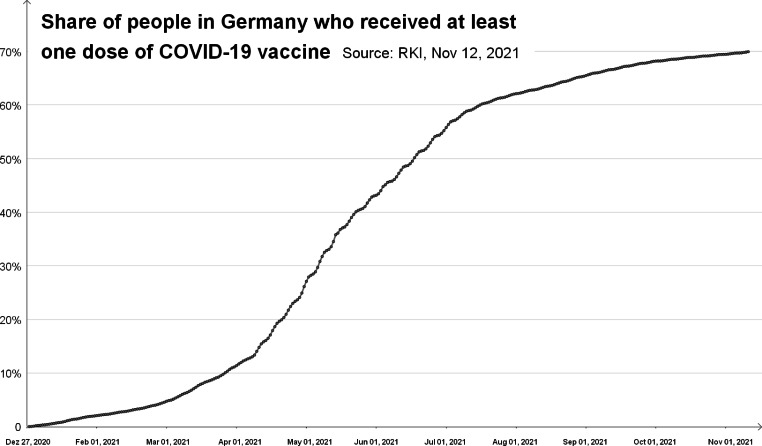
Share of people in Germany who received at least one dose of Covid-19 vaccine (December 2020–November 2021)

In Fig. 4, a steep exponential increase can at first be seen followed by a clear slowdown from March and then a transition into an S-curve. We find such an S-curve not only in the cumulative number of vaccinations, but also in the number of infections (e.g. Figure 3).

What type of growth is present is difficult to discern from a graphical presentation and often depends on the time and duration from which data are considered. For example, a curve that rises quite steeply in a particular area may be due to a high degree of polynomial growth. With respect to time-limited sections, students may also get the idea that it is linear growth. In terms of mathematical modelling, an opportunity is provided to discuss with students the appropriateness of different mathematical models and the dependence of the chosen model on contextual knowledge.

**Fig. 5:**
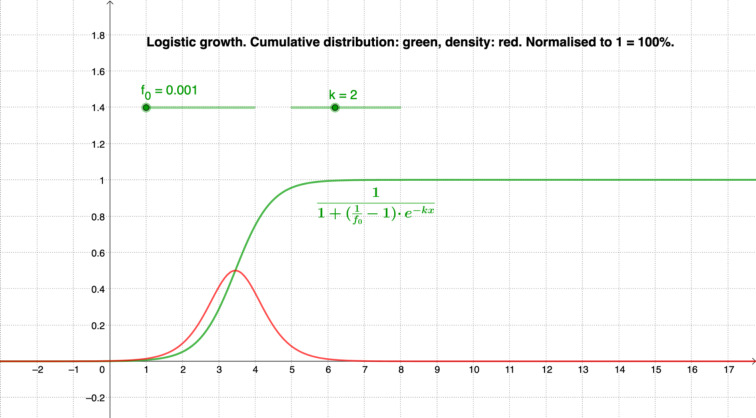
Cumulative S-shaped distribution (in green) and associated density distribution (in red) (Elschenbroich, 2020)

Especially significant for the case presented in Fig. 4 is basic mental model 1, namely that growth by the same factor takes place in the same unit. The notion of an S-curve showing so-called logistic growth, which combines exponential growth (at the beginning) and limited growth, is the curve of *natural* growth, which is significant here. In addition to the representation of the infection event as a *cumulative* distribution, which naturally does not decrease, this is very much the case with the bell-shaped *density distribution* (cf. Figure 5). In the first phase of exponential growth, both the density distribution (new infections, red line in Fig. 5) and the cumulative distribution (total infections, green line in Fig. 5) increase exponentially. This is due to the properties of exponential functions when integrating or differentiating and to the fact that the density function is the derivative of the cumulative distribution function. Here, the exponential decrease occurs only in the density, that is, in the new infections.

**Fig. 6:**
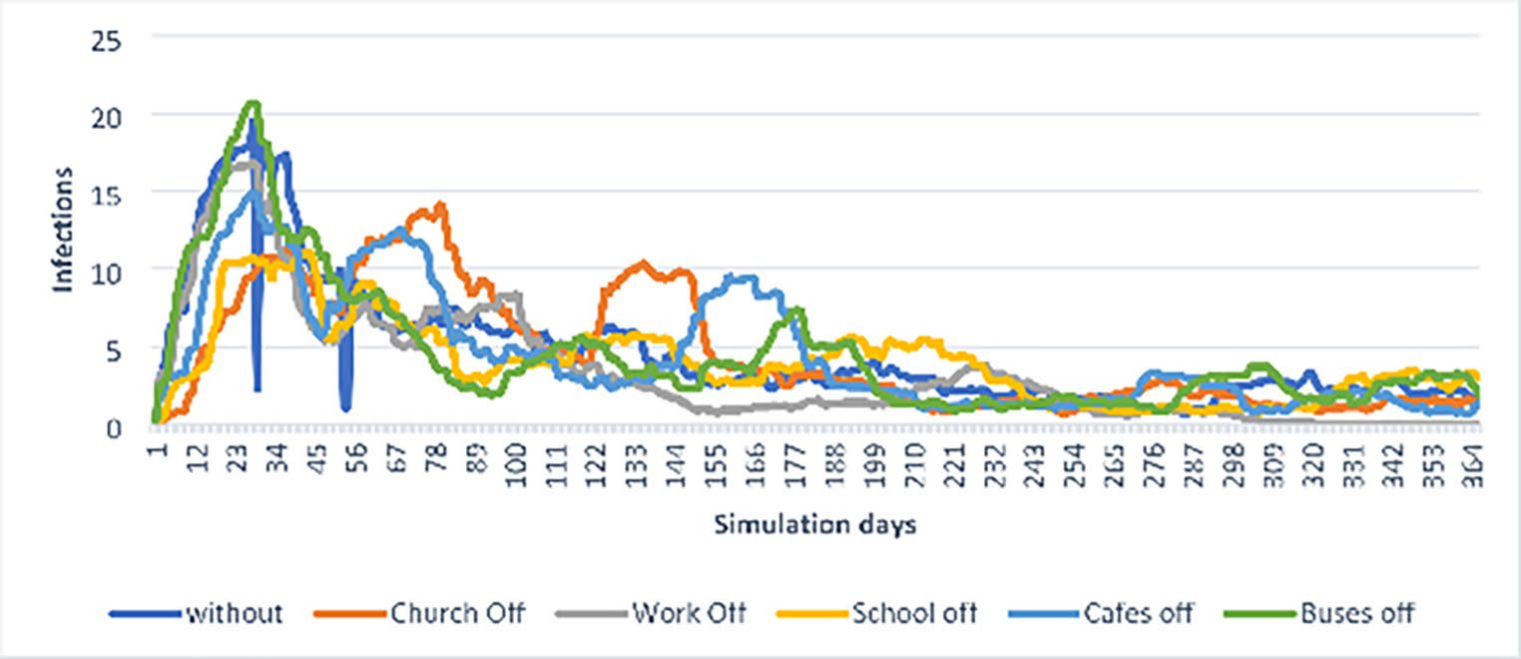
Predictive modelling by comparatively analysing effects. Graphic provided by students of the Gymnasium Lerchenfeld *(and we thank them for providing the image)*

In the cumulative distribution, there is still an increase because the number of total infections will never decrease—becoming zero at best, but never negative. In other words, if the increase increases exponentially, the accumulation also increases exponentially. If the increase falls exponentially, the accumulation stabilizes. This relationship can be displayed dynamically for different parameters, for example as shown in Fig. 6, in which the influence of the relevant parameters (f_0_ and k) can be shown by sliders.

Through such an approach, learners can experience that the choice of a mathematical model depends in some situations not only on the real given data but also on the knowledge of a specific context. Such a procedure enables them to work out two of the four basic mental models closely related to the context, as follows:

- Identifying exponential growth analytically as growth proportional to the current stock, that is to recognise that growth by the same factor occurs in the same (time) unit.
- Detecting an increasingly steep increase in the stock curve and thus to infer exponential growth.

At the same time, learners not only gain a deeper understanding of exponential growth itself but also learn to distinguish it from other growth processes, such as linear growth, not only symbolically but also analytically. Additionally, they learn that a graphical representation is not always suitable for determining the nature of growth without further knowledge of the context or consideration of a long period of time.

Related to mathematical modelling, students concurrently experience how the choice of model influences the achieved results, and therefore has to be considered carefully in terms of how far the mathematical model correctly reflects the part of the reality that should be represented. With the help of these problems, it can be made clear to students that they are fundamentally capable of dealing with such questions; they do not simply have to accept statements about growth processes reported in the media as a given but can check them independently. This was and is important insofar as politicians (usually with the support of scientists) have used the nature of growth to justify the introduction of certain measures. We discuss this aspect in more detail in a second example.

### Modelling of the course of infection under different assumptions

Since the beginning of the pandemic, various models have been established with different objectives. These include explanatory models that have been used to explain why certain measures are necessary, for example to avoid overburdening health systems. In addition, predictive models have often been presented to show the development of the incidence of infection under the influence of different measures. This can be used in mathematics lessons as a starting point to discuss which content-related assumptions underlie the given graphs.

Regarding explanatory models, reference can be made to graphs from the beginning of the pandemic. As there were no vaccines at the time, and none were in sight, models usually assumed that the number of infected people (i.e. the integral over the density distribution) would remain constant in the long term and would only be distributed differently over a longer period until herd immunity had been achieved. The aim was to ‘flatten the curve’ through appropriate interventions (lockdown, hygiene measures) so that healthcare systems would not be overburdened during peaks of infection.

Predictive models can be set up to contrast the impact of different measures (or a combination of them). One example would be to theme plots showing the impact of different types of lockdown on daily new infections and bed occupancy in intensive care units. Another option would be to have students create such plots themselves using simulation software. Here, students can make different assumptions, such as the duration of simulations, the number of people in the model, the type of institutions and organisations available (e.g., schools, restaurants, public transportation, workplaces, churches) and assumptions about hygiene measures (e.g., quarantine regulations, minimum distance) and the infection rate itself. Subsequently, it might be determined, for example, which organisations and institutions would be closed, and the students could interpret the effects comparatively (cf. Figure 6).

Such predictive models are therefore always dependent on the assumptions that are incorporated into them, and therefore represent forecasts for future developments.

Forecasts about the growth of infection numbers are in some ways comparable to weather forecasts: over a few days, they are relatively reliable, but over a longer period, they become increasingly unreliable. In Germany, a forecast made by the RKI^1^ on March 13, 2021, which predicted an incidence of around 350 for April 15, 2021, was especially criticised. In fact, the incidence was 162.

Even if a grey confidence funnel were placed around the extrapolated trend line, the minimum predicted value would be far higher than the actual values achieved, even at the lower edge. This graph can be used to discuss the influence of certain values on the results of modelling. Among other things, the assumption of parameters used being stable over a longer period of time, is not secure and can be addressed in mathematics classrooms. By contrast, in the event of such a rapid increase, massive political measures would have to be expected (e.g., hard lockdown, compulsory vaccination), which would have a considerable influence on further developments. By using simulation software or plots of the influence of different parameters on the course of infection, students can understand the impact (on the models’ results) of changing the underlying assumptions, without having to model the infection completely themselves.

In terms of the modelling process, the focus on addressing such questions is on the assumptions made in steps 1 and 2 of the modelling process when understanding and constructing the real situation as well as simplifying and structuring the situation model, as shown in Fig. 1, not so much on identifying the extent to which the results depend not only on the model chosen (such as in Sect. 3.1). Students can learn how important it is for the evaluation of individual models to know the model assumptions and critically to question their appropriateness. Again, this applies not only to autonomous modelling but also, as in the case shown, to situations in which decisions have been made on the basis of modelling. Students can thus experience, as in the example above, that they can and should question such modelling as responsible citizens. In this example, such questioning is not with regard to the selected model, but with regard to the question of whether the selected influencing factors describe the situation adequately.

In the context of the concept of exponential growth, the examples in this section show that the analysis of different models of infection incidence, more specifically the analysis of different influencing factors to be considered (see example 1) as well as the choice of growth factor (see example 2) to contain infection incidence, can contribute to the need for the following basic mental models:

- Exponential growth symbolically as ${N}_{new}={N}_{new-1}+Increment$ with $Increment \propto {N}_{new-1}$ to hold on to.
- To recognise the growth factor as a ratio $\frac{\varDelta {N}_{new}}{\varDelta {N}_{new-1}}$.

It should be recognised that exponential growth occurs by the same factor in the same unit, and the growth factor must remain approximately constant during exponential growth.

**Fig. 7:**
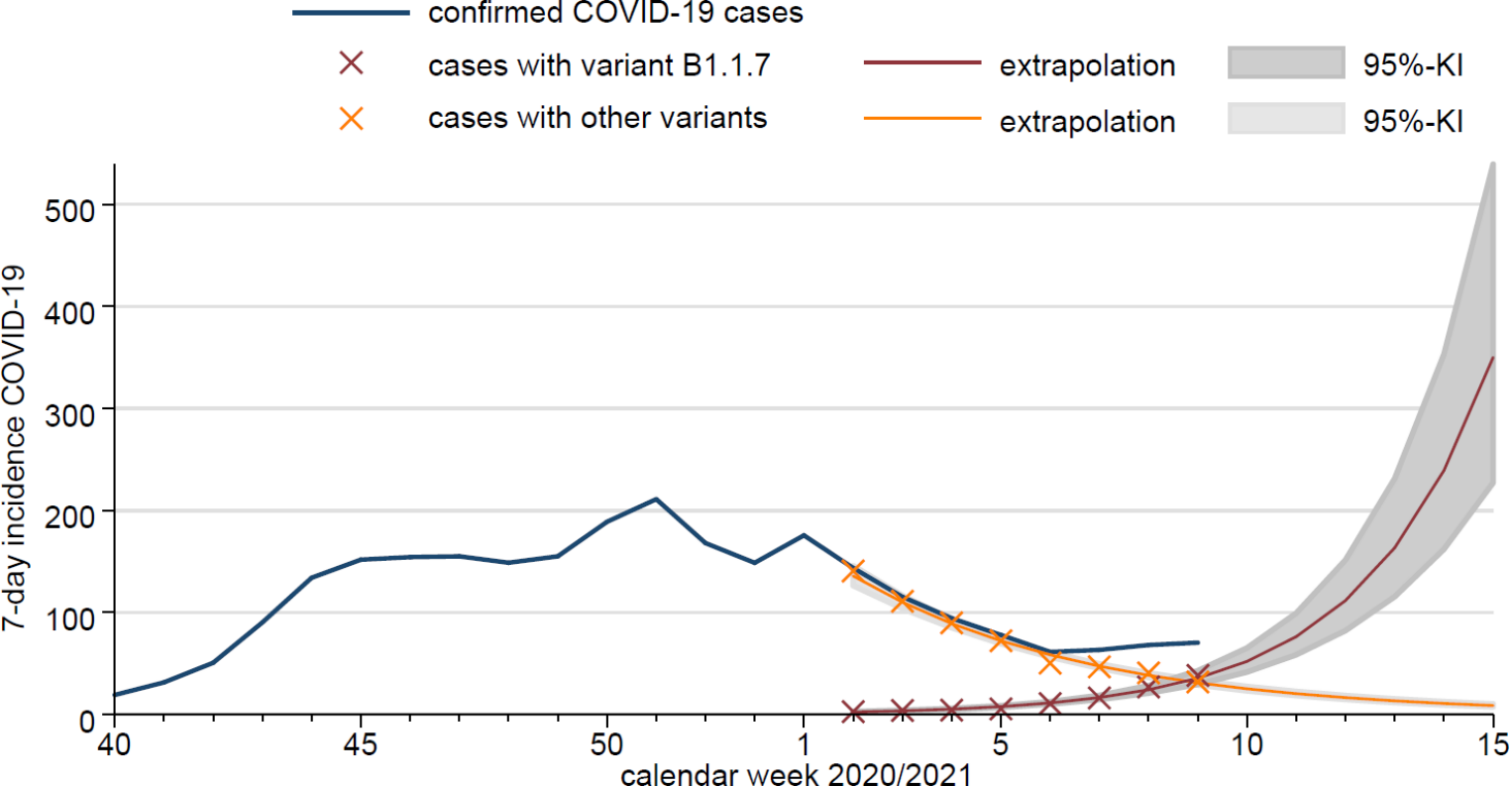
RKI forecast from March 13, 2021 (RKI 2021, https://www.rki.de/DE/Content/InfAZ/N/Neuartiges_Coronavirus/Situationsberichte/Maerz_2021/2021-03-13-de.pdf?__blob=publicationFile). Daily situation report of the RKI on coronavirus disease-2019. (Accessed May 27, 2021)

### Determination of the exponential factor at different times

**Fig. 8:**
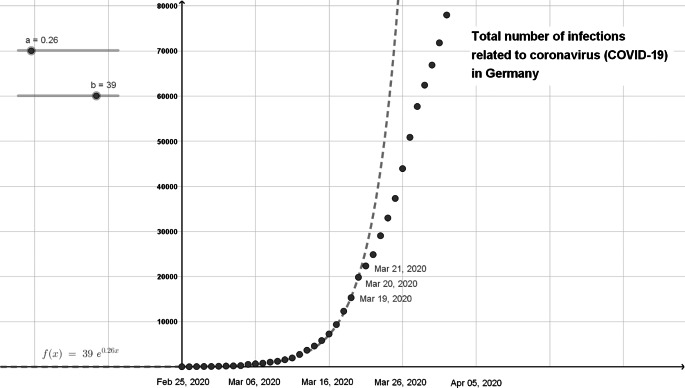
After varying the parameters on slider a accordingly, it can be seen that for a = 0.26, exponential growth took place in Germany up to March 21, 2020 (Elschenbroich, 2020)

The data reported daily during the pandemic provide a modelling context for exponential or logarithmic growth processes, since real data can be examined experimentally to determine whether exponential growth existed in the past, and if so, for how long (see Fig. 8). For mathematics lessons, the question can therefore be formulated as to how the exponential factor can be calculated for a limited period of time, and how it is related to the R-value given in the media on a daily basis. The importance of the meaning of this value can be indicated as a deterministic (albeit never numerically precise) parameter. The calculation of it has a mathematical explanation (Murray, 2008, p. 322), even in mathematics classrooms settings.

In addition to calculating the doubling time, challenges also lie in the model’s assumptions as a means to enable influences on exponential growth, such as the growth factor, to be describable. The relevant and interesting question is how long it takes for a doubling to take place and how daily growth factors can be calculated.

In a large German state, it can be seen by way of example that there was roughly a doubling from March 15, 2020, with 2176 cases, to March 18, 2020, with 4366. Thus, the doubling time was pretty much exactly three days. The daily growth factor was therefore $\sqrt[3]{2}$ ≈ 1.26 in general, and n days’ doubling time the daily growth factor q = $\sqrt[n]{2}$.

Nevertheless, one does not always have ‘clean’ doubling in the data, but the information that in d days, the value of a has increased to b, and from this, one can calculate precisely the doubling time D. The equation $b=a\bullet {2}^{x}$ generates the solution $x=\frac{\text{ln}\left(b\right)-\text{l}\text{n}\left(a\right)}{\text{l}\text{n}\left(2\right)}$, and thus $d=D\bullet x$. From this follows D = $\frac{d}{\frac{\text{ln}\left(b\right)-\text{l}\text{n}\left(a\right)}{\text{l}\text{n}\left(2\right)}}=\frac{d\bullet \text{l}\text{n}\left(2\right)}{\text{ln}\left(b\right)-\text{l}\text{n}\left(a\right)}$ .

The effects of shortening the doubling time are usually seriously underestimated. How much will a stock grow in one month (= 30 days) if the doubling time is known?

- Doubling every 30 days, it will increase by a factor of 2.
- Doubling every 15 days, it will increase by a factor of 2^2^ = 4.
- ….
- Doubling every day, it will increase by a factor of 2^30^ = 1,073,741,824.

This fact, which is not very complicated from a purely mathematical point of view, is difficult to understand regarding the Covid-19 pandemic in that the stock size is the number of infected persons. It is this stock size that also determines growth of infections, since a larger stock size also implies a larger growth rate. From a mathematical point of view, therefore, it is not surprising that the growth factor, which is repeatedly referred to in the media as R-value (for reproduction value) is such a relevant parameter. In general discussion, the R-value is regarded as the daily infection rate. However, this is inaccurate. The reproduction value R indicates how many persons are infected on statistical average by an infectious person within the so-called *generation time*. With an R-value of 1.3 on a generation time of 4 days, we thus have a daily growth factor of $\sqrt[4]{1.3}$ ≈ 1.068.

The R-value is an epidemiological indicator to describe the dynamics of an outbreak. If it is above 1 for a longer period, the number of infections increases; if it is below 1 for a longer period, the number of infections decreases.

**Fig. 9:**
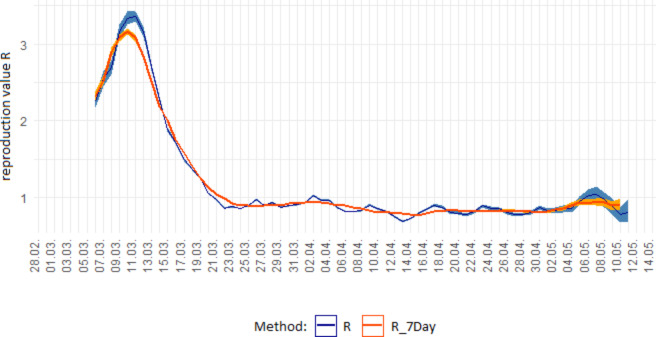
Comparison of 4-day R-value and 7-day R-value. (https://www.rki.de/DE/Content/InfAZ/N/Neuartiges_Coronavirus/Projekte_RKI/R-Wert-Erlaeuterung.pdf?__blob=publicationFile)

Meanwhile, in addition to this 4-day R-value, there is also the 7-day R-value. This is a more stable, smoothed-out version of the R-value, since it balances out the fluctuations due to weekend effects by using the weekly period (Fig. 9).

**Fig. 10:**
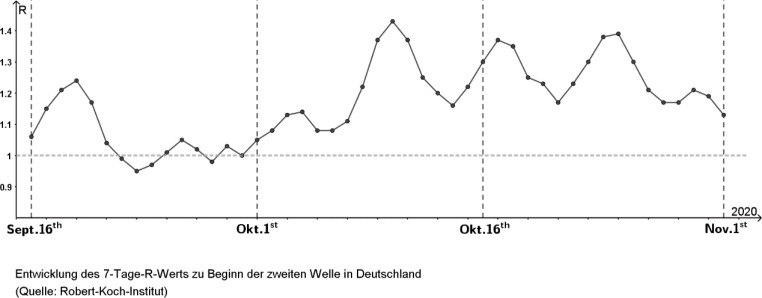
Development of 7-day R-value based on daily reported new infections at the beginning of the second wave in Germany. (Source: Robert Koch Institute)

In each case, the R-value is calculated as the quotient of the respective summed new infection numbers during each period and assigned to the last day (see Fig. 10).

If the R-value (for either 4 or 7 days) were constant over a longer period, this would result in a ‘clean’ exponential growth. However, it is precisely the goal of health policy to avoid or stop exponential growth and to lower the R-value, if possible, to well below 1. For this reason, the observed infection figures only showed a truly exponential growth in the short term during the beginning of the Covid-19 pandemic.

Examining this R-value, however, brings with it the difficulty that in the (application) context of the Covid-19 pandemic, the value would vary—albeit sometimes only slightly—in successive counting periods, thus failing to satisfy a central property of the growth factor of the exponential function: quotient equality $\frac{\varDelta {N}_{new}}{\varDelta {N}_{new-1}}$. Therefore, the question arises as to the appropriate mathematical model and possible simplifications.

**Fig. 11:**
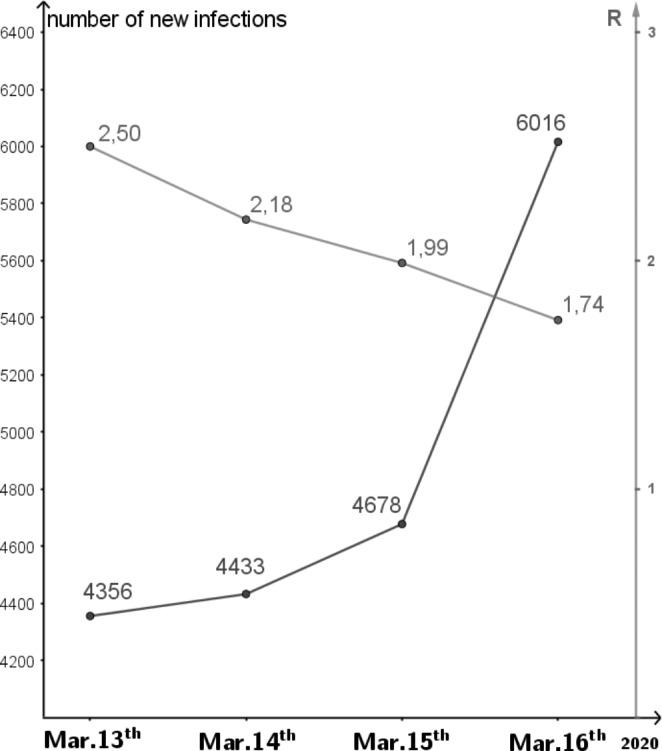
Estimation of R-value and results of nowcasting during the period of March 13–16, 2020. (Source: Robert Koch Institute, data as of June 15, 2020)

For example, if we consider the new infection figures (taking into account the nowcasting results of the Robert Koch Institute^2^, where the actual new infection figures per day are estimated on the basis of constantly updated data), this is in contrast with the reported new infection figures, which only increase when a positive test result is obtained and do not include the number of unreported cases. At the start of the pandemic in Germany, the R-value was 2.50 on March 13, 2020, and it fell to 1.74 over the following days (Fig. 11).

When examining the central characteristic property of exponential growth in the development of infection numbers at the beginning of the pandemic, a ‘trend line’ or similar would therefore be more suitable and even necessary to disregard deviations of the R-value. It is very probable in the context of reality, but irrelevant for the application-related interpretation as being ‘exponentially growing’. This distinguishes the investigation of growth in the context of the Covid-19 pandemic from common curricular standard examples. Even a focus on the 4- or 7-day R-values mentioned above does not compensate for this.

Another observation from the discussion on infection figures and the pandemic-related debate on exponential growth processes is the initial focus on single, isolated figures, such as the separate consideration of new infection figures and R-value. The relevant question in this context refers to what is growing exponentially: is it the overall infection numbers or the new infection numbers? While classical examples such as the development of bacterial cultures and cooling processes tending to look at the change in stock (i.e., the specific number of bacteria at a specific point in time or the specific temperature at a specific point in time), in the case of the pandemic, the public focus has primarily been on new infection figures. Exponential growth thus refers to a *change* in a population. It is therefore essential to consider the R-value and the new infection figures in combination in order to capture characteristic properties of exponential growth processes and be able to assess the infection situation.

It can be concluded that the stock of Covid-19 infections, that is, the number of people already or currently infected, is also increasing exponentially. In the public discussion, however, this figure plays an increasingly minor role. This makes little sense in any case, if vaccination against the virus is successful and, moreover, knowledge is available regarding possible multiple infections through virus variants. Concerning the distinction between stock and change, questions relating to the other examples mentioned are usually more one-dimensional.

In summary, the notion of proportionality of increment and current stock is central as a notion for exponential growth, that is, that growth occurs by the same factor in the same unit. Thus, students find that caution, as illustrated, is needed when considering the R-value in relation to the notion of quotient equality $\frac{\varDelta {N}_{new}}{\varDelta {N}_{new-1}}$: since we have calculated it from daily fluctuating values, quotient equality is not certain. In this way, students can learn how strong the influence of the growth factor is on the result obtained. This can lead to the realisation that long-term predictions should always be viewed with caution, as even a small change in growth factor can lead to significant differences in outcome. In relation to mathematical modelling, students can thus learn about the importance of considering what specific values are used for variables (in this example, the question of 4- and 7-day R-values).

The uncertainties about and problems in determining a meaningful growth factor should not lead to the fact that this question is not addressed in school mathematics lessons. On the contrary, in the sense of general education, this point should be openly discussed with students. Only by doing so, students can gain, not just the opportunity to learn to independently question the information presented in the media, but also experience of how this information is generated and to what extent it may be problematic.

## Discussion

A look at discussions on mathematics education shows that growth processes have always been considered to be very relevant content (see Sect. 3.1 & 3.2) by different authors (Ärlebäck et al., 2013; Confrey & Smith, 1994; Ellis et al., 2015), and they should have special importance in the curriculum, especially because of their central role of growth processes in everyday life. The experience of the pandemic has highlighted the importance of this discussion and made it clear that an understanding of exponential growth is central to understanding and assessing current developments that have a direct influence on our daily lives. At the same time, the Covid-19 pandemic has provided numerous occasions for students to engage in modelling problems from this context in secondary school mathematics (Kwon et al., 2021). One possibility is to let the students themselves make predictions about possible developments based on existing data (Heyd-Metzuyanim et al., 2021). Another way for students to become aware that they can and should question media reports themselves is to analyse such reports with regard to the data and models used in them (Aguilar & Castaneda, 2021), which is the way that we have chosen in this paper.

The analysis of the examples presented, namely modelling the course of infection by using different models and assumptions (see Sect. 4.1 & 4.2) and determining the exponential factor at different times (see Sect. 4.3) here shows that exponential growth should play an important role in teaching, not only in the sense of applying calculations, such as by determining growth factors but also in relation to building appropriate basic mental models, such as imagining growth proportional to the current stock (Castillo-Garsow, 2013; Sezer & Namukasa, 2021). First, students need to define the goal of modelling; for example, should an existing model, which is only represented by a graph, be questioned regarding the appropriateness of the representation of a real situation? Are they to reconstruct which mitigation measures were chosen based on the modelling presented, contrast different measures taken and assess the reasonableness of the choice of mitigation measures?

Second, when transferring the real model into a mathematical model, the discussion of a growth model and the data or values to be chosen are particularly important for contexts surrounding the Covid-19 pandemic. In addition to choosing an appropriate growth model, it is also important to determine the data that should be used for the modelling. Thought should be given to the source of the data and the time interval that should be used.

Furthermore, the analysis shows that different basic mental models are supportive for understanding the various central questions and mathematical approaches surrounding the pandemic. For example, we were able to work out that prototypes of graphical ideas about exponential growth are useful and necessary to be able to read from given courses whereby infections have increased exponentially. However, basic mental models concerning other types of growth are helpful in order to be able to question critically whether the chosen model can meaningfully fit the available information in a given period.

However, not only is the content relevant for a deeper understanding of exponential growth (Rotem & Ayalon, 2021), but so is the competent application of the content in terms of mathematical modelling. Modelling works with assumptions and inevitably with simplifications. The more parameters are included in modelling, the more realistic—but also more complex—the model becomes. If sound basic mental models about exponential growth are available, they can enable students to become aware of the problems of this functional class. This can lead them to make more careful and conscious decisions about complexity reduction, and thus about appropriate modelling, or to precisely identify these aspects more easily from the modelling shown. The broad use of basic mental models for specific mathematical content leads to decisions being made about complexity reduction or more appropriate modelling when post-modelling—in this case the recognition of exponential growth. Another important aspect of modelling is validation. This requires knowing different models and being able to check the plausibility of the results. Together, this shows that the pandemic-context is complex from a mathematical point of view and must be considered holistically. In order to question the representations used in the media and understand the concepts behind them, basic mental models about exponential growth are necessary. However, the examples presented also show that even very complex relevant problems can be dealt with in sufficient depth in mathematics education. Thus, from the age of about fourteen, (in Germany students from grade 9 onwards) students are able to understand social-relevant events and participate responsibly in society.

Modelling includes not only forecasts with concrete data but also dealing with the creation of mathematical models and the results of them. This includes several modelling runs in which the model is checked for its usefulness on the basis of the actual development and adjusted if necessary. Simple models are usually not particularly reliable in their forecasts, but many are often important for understanding a social or natural real-life situation. Therefore, they are not to be immediately discarded. Sophisticated modelling, however, is so complex that it is now no longer verifiable, neither by laypeople nor also by politicians, who must rely on the results of modelling to inform their political decisions. In social and political discourse, this can lead to a preference for modelling that is more in line with one’s own views and opinions, either because one is closer to the people conducting modelling or because the results are more in line with one’s own world view. One must always keep the purpose of the modelling in mind. For example, does the number of hospital places need to be estimated upwards, or can the number of pupils who can still come to school in good health be determined? Transparent disclosure of the modelling purpose is important in modelling processes.

By falling back on basic mental models, as shown in our contribution, the argumentation can, if necessary, be conducted more rationally, since knowledge about the context (as shown in Sect. 3.1) is necessary to model growth in a meaningful way. The subjective point of view recedes into the background, and the world orientation gains in importance from a mathematical perspective.

Especially when referring to current authentic problems such as COVID-19, modelling competence plays a special role. Questions, and ultimately goals, must be defined that are to be answered with the respective model, as well as corresponding simplifications discussed, mathematisations made and results interpreted. Finally, the validation of the results and model are of particular importance when the results of the modelling have an impact on people’s lives, as became clear in Sect. 3.2. A particularly important aspect in connection with the Covid-19 pandemic is that experts’ predictions often did not come true. This also depends on the corresponding model assumptions, which should be disclosed. Understanding that such models may not be accurate is an important lesson to learn from the modelling context, even for experts.

The discussion on how to deal with the Covid-19 pandemic has highlighted the significant importance of mathematical knowledge, which includes not only the correct application of calculus but also the corresponding ideas and basic mental models, as well the ability to model and knowledge of modelling processes (Lammers et al., 2020). A (future) responsible citizen needs all these facets in terms of general education. The context serves the understanding of exponential growth, and this understanding is necessary for being able to act responsibly in crises. Consequently, the assessment of content in standards and curricula has to be constantly questioned and supplemented against a background of the required general educational contents, basic mental models and process-related competences. After the ‘PISA shock’ in 2000, the Covid-19 pandemic has now brought renewed impetus for a corresponding further development to include missing aspects and their interlinking.

It becomes clear that basic mental models on exponential growth and mathematical modelling in this connection are the prerequisites for overcoming the current challenges.

## Conclusion

Forecasts, obtained through mathematical modelling, do indeed serve as a basis for political decision-making time and again, usually with the aim of preventing the occurrence of undesirable predictions. In the case of the hard lockdown, we were able to experience this decision-making at first hand in many countries in 2021. Whereas with ‘non-critical’ models, we can wait calmly to see what development will occur and make one or more modelling runs, this has not been possible with either climate development or the Covid-19 pandemic. The fact that there have also been incorrect forecasts in model calculations for the pandemic is not surprising, but rather to be expected. The force with which Covid-19 affected society as a whole and some sectors and groups of people in particular has not only made it difficult for politicians to make consistent but appropriate decisions based on uncertain facts, but also led to opposition and protests because of a partial lack of social acceptance. By these examples, students experience that elementary mathematical methods and concepts serve as a basis for discussion in political decision-making, but that their use must be critically questioned with regard to their appropriateness.

Knowledge about the possibilities and limitations as well as factors influencing the results of modelling can also help to understand developments that do not meet expectations because the model assumptions originally made later turned out to be unrealistic. Overall, however, an understanding of the models used is a prerequisite for being able to work in an appropriately mathematical way, both in terms of content and process. Here, too, a sound understanding of basic mental models can in turn help to counteract such currents.

In mathematics classrooms, it will not be possible to address modelling at a level that has been developed over months in relevant scientific institutes. Only simpler and inevitably under-complex models can be dealt with here. With the help of our examples, it can already be shown to learners what added value mathematical modelling based on authentic data leads to initial results. But in any case, the problems of mathematical modelling and the necessity of several modelling runs can and must be discussed. Basic mental models help to question the quality of generated results and, above all, to understand basic properties of exponential growth, also in comparison with other types of growth. In our contribution we clearly show how basic mental models at the transition between reality and mathematics help to generate knowledge about models in general and about exponential growth in particular, in order to strengthen the necessary progressions or learning phases of the students. In this way, it also becomes clear why the explicit basic mental models about exponential growth should be consistently developed in mathematics lessons; however, this should not only be done through modelling scenarios but also in pure mathematics.

Nevertheless, it must be clear that the result of a model does not mean that reality will develop in this way. Rather, it cannot be concluded from the non-occurrence of a forecast that it was wrong. After all, measures may have been taken because of the forecast precisely to prevent the forecast from being realised (e.g., lockdown to prevent the collapse of the healthcare system).

Also, model parameters must remain manageable. In this respect, one could say pointedly that modelling in school can only be a model of modelling, but a model on which the principles of modelling can be learned and understood and which must be connected to content-related ideas. However, it is important to impart this knowledge in order to give citizens the opportunity to have a say in society.

A different situation arises when such modelling is not carried out as part of individual school lessons, but as part of project days in which students work on a complex problem over a longer period of time. This form of integrating modelling into the classroom has a long tradition in German-speaking countries (Humenberger & Siller, 2022; Kaiser et al., 2013; Vorhölter et al., 2019). Under such circumstances, students can perform complex modelling even on the basis of given infection numbers, that is, large amounts of data over a longer period of time. For this purpose, they can either be given concrete questions that help them to analyse the given data (e.g., when the increase in infections was highest, to what extent can the influence a particular containment measure had can be reconstructed retrospectively from the data; likewise, how a future course can be forecast), or they develop questions of interest themselves, which they then work on.

The importance of understanding exponential growth for modelling the Covid-19 pandemic, as well as considering its real-life implications, is not only an important point of connection in current mathematics education but it should also be considered with a view to future educational standards. In this, the reference to the understanding of different types of growth should be made more strongly, even in lower grades, and at the same time the modelling competence, especially with reference to the modelling of data, should be emphasised more strongly. This issue also points to possible failures in mathematics education in the past. In our view, it is crucial that different approaches to learning and understanding exponential growth are subjected to evidence-based investigation in terms of their impact on students and their learning effectiveness. These can be an interplay of basic mental models and mathematical modelling, as outlined in our paper, or focus on exponential growth as such (Da Silva et al., 2021; Krause et al., 2021).

Our paper shows possibilities of implementing a very current topic in the classroom from a theoretical-foundational and epistemological perspective. A next step is the detailed investigation of the implementation with students in mathematics lessons. In particular, it should be determined which effects the use of necessary basic mental models in modelling a complex real situation with the help of mathematics has on the effectiveness of students’ mathematical understanding, the motivation of the participants as well as the understanding of the participants.

## Footnotes

1 The Robert Koch Institute (RKI) is the German government’s central scientific institution in the field of biomedicine.

2 The Robert Koch Institute is the biomedical lead research institution of the German Federal Government.

## References

[CR1] Aguilar MS, Castaneda A (2021). What mathematical competencies does a citizen need to interpret Mexico’s official information about the COVID-19 pandemic?. Educational Studies in Mathematics.

[CR2] Ärlebäck JB, Doerr HM, O’Neil AH (2013). A modeling perspective on interpreting rates of change in context. Mathematical Thinking and Learning.

[CR3] Bakker A, Cai J, Zenger L (2021). Future themes of mathematics education research: An international survey before and during the pandemic. Educational Studies in Mathematics.

[CR4] Blomhøj, M. (2004). Mathematical modelling: A theory for practice. In B. Clarke, D. M. Clarke, G. Emanuelsson, B. Johansson, D. V. Lester, A. Wallby, & K. Wallby (Eds.), *International Perspectives on Learning and Teaching Mathematics* (pp. 145–159). National Center for Mathematics Education

[CR5] Blomhøj M, Jensen TH (2003). Developing mathematical modelling competence: Conceptual clarification and educational planning. Teaching Mathematics and Its Applications.

[CR6] Blum, W., & Leiß, D. (2007). How do students and teachers deal with modelling problems? In C. R. Haines, P. L. Galbraith, W. Blum, & S. Khan (Eds.), *Mathematical Modelling (ICTMA 12): Education, Engineering and Economics* (pp. 222–231). Horwood. 10.1533/9780857099419.5.221

[CR7] Blum W, Niss M (1991). Applied mathematical problem solving, modelling, applications, and links to other subjects—State, trends and issues in mathematics instruction. Educational Studies in Mathematics.

[CR8] Bush SB, Gibbons K, Karp KS, Dillon F (2015). Epidemics, exponential functions, and modeling. Mathematics Teaching in the Middle School.

[CR9] Castillo-Garsow C (2013). The role of multiple modeling perspectives in students’ learning of exponential growth. Mathematical Biosciences and Engineering.

[CR10] Confrey J, Smith E (1994). Exponential functions, rates of change, and the multiplicative unit. Educational Studies in Mathematics.

[CR11] Da Silva, A. S., Barbosa, M. T. S., de Souza Velasque, L., da, Alves, S. B., D., & Magalhães, M. N. (2021). The COVID-19 epidemic in Brazil: How statistics education may contribute to unravel the reality behind the charts. *Educational Studies in Mathematics*, *108*(1–2), 269–289. 10.1007/s10649-021-10112-6 10.1007/s10649-021-10112-6 PMC8514808 34934244

[CR12] De Bock D, van Dooren W, Janssens D, Verschaffel L (2002). Improper use of linear reasoning: An in-depth study of the nature and the irresistibility of secondary school students’ errors. Educational Studies in Mathematics.

[CR13] Ellis AB, Özgür Z, Kulow T, Williams CC, Amidon J (2015). Quantifying exponential growth: Three conceptual shifts in coordinating multiplicative and additive growth. The Journal of Mathematical Behavior.

[CR14] Elschenbroich HJ (2020). Mathematik und Corona-Infektionen. MNU Journal.

[CR15] Engelbrecht J, Borba MC, Llinares S, Kaiser G (2020). Will 2020 be remembered as the year in which education was changed?. ZDM – Mathematics Education.

[CR16] Gal, I., & Geiger, V. (2022). Welcome to the era of vague news: A study of the demands of statistical and mathematical products in the COVID-19 pandemic media. Educational Studies in Mathematics. 10.1007/s10649-022-10151-7 10.1007/s10649-022-10151-7 PMC9036505 35496813

[CR17] Greefrath, G., & Vorhölter, K. (2016). *Teaching and Learning Mathematical Modelling. Approaches and Developments from German Speaking Countries*. Springer International Publishing. 10.1007/978-3-319-45004-9

[CR18] Hefendehl-Hebeker, L., Hofe, V., Büchter, R., Humenberger, A., Schulz, H., A., & Wartha, S. (2019). Subject-matter didactics. In H. N. Jahnke & L. Hefendehl-Hebeker (Eds.), *Traditions in German-Speaking Mathematics Education Research* (pp. 25–59). Springer International Publishing. 10.1007/978-3-030-11069-7_2

[CR19] Heyd-Metzuyanim E, Sharon AJ, Baram-Tsabari A (2021). Mathematical media literacy in the COVID-19 pandemic and its relation to school mathematics education. Educational Studies in Mathematics.

[CR20] Humenberger, H., & Siller, H. S. (2022). *Modelling Activities in German Speaking Countries, Modelling in Science Education and Learning, 15(1)*. https://polipapers.upv.es/index.php/MSEL/issue/view/1133

[CR21] Hußmann S, Prediger S (2016). Specifying and structuring mathematical topics: A four-level approach for combining formal, semantic, concrete, and empirical levels exemplified for exponential growth. Journal Für Mathematik-Didaktik.

[CR22] Hutzler F, Richlan F, Leitner MC, Schuster S, Braun M, Hawelka S (2021). Anticipating trajectories of exponential growth. Royal Society Open Science.

[CR23] Kaiser, G. (2007). Modelling and Modelling Competencies in School. In C. R. Haines, P. L. Galbraith, W. Blum, & S. Khan (Eds.), *Mathematical Modelling (ICTMA 12): Education, Engineering and Economics* (pp. 110–119). Horwood. 10.1533/9780857099419.3.110

[CR24] Kaiser, G. (2020). Mathematical modelling and applications in education. In S. Lerman (Ed.), *Encyclopedia of Mathematics Education* (pp. 553–561). Springer International Publishing. 10.1007/978-3-030-15789-0_101

[CR25] Kaiser, G., Bracke, M., Göttlich, S., & Kaland, C. (2013). Authentic complex modelling problems in mathematics education. In A. Damlamian, J. F. Rodrigues, & R. Sträßer (Eds.), *Educational Interfaces Between Mathematics and Industry* (Vol. 16, pp. 287–297). Springer International Publishing. 10.1007/978-3-319-02270-3_29

[CR26] Kaiser, G., & Brand, S. (2015). Modelling competencies: Past development and further perspectives. In G. Stillman, W. Blum, & M. Salett Biembengut (Eds.), *Mathematical Modelling in Education Research and Practice* (pp. 129–149). Springer International Publishing. 10.1007/978-3-319-18272-8_10

[CR27] Keren G (1983). Cultural differences in the misperception of exponential growth. Perception & Psychophysics.

[CR28] Kleine M, Jordan A, Harvey E (2005). With a focus on ‘Grundvorstellungen’ Part 1: A theoretical integration into current concepts. ZDM – Mathematics Education.

[CR29] Krause CM, Di Martino P, Moschkovich JN (2021). Tales from three countries: Reflections during COVID-19 for mathematical education in the future. Educational Studies in Mathematics.

[CR30] Kwon ON, Han C, Lee C, Lee K, Kim K, Jo G, Yoon G (2021). Graphs in the COVID-19 news: A mathematics audit of newspapers in Korea. Educational Studies in Mathematics.

[CR31] Lammers, J., Crusius, J., & Gast, A. (2020). Correcting misperceptions of exponential coronavirus growth increases support for social distancing. *Proceedings of the National Academy of Sciences*, *117*(28), 16264–16266. 10.1073/pnas.2006048117 10.1073/pnas.2006048117 PMC7368332 32581118

[CR32] Lesh R (2010). Tools, researchable issues & conjectures for investigating what it means to understand statistics (or other topics) meaningfully. Journal of Mathematical Modeling and Application.

[CR33] Levy MR, Tasoff J (2017). Exponential-growth bias and overconfidence. Journal of Economic Psychology.

[CR35] Maaß, K. (2006). What are modelling competencies? *ZDM*, *38*(2), 113–142. 10.1007/BF02655885

[CR36] Murray, J. D. (2008). *Mathematical biology. 1: An introduction* (5.). Springer. corr. print)

[CR37] Niss, M., Blum, W., & Galbraith, P. L. (2007). Introduction. In W. Blum, P. L. Galbraith, H.-W. Henn, & M. Niss (Eds.), *Modelling and Applications in Mathematics Education. The 14th ICMI study* (pp. 3–32). Springer US. 10.1007/978-0-387-29822-1_1

[CR38] Rotem SH, Ayalon M (2021). Exploring Israeli high school graduates’ explanations for the spread of the coronavirus. Educational Studies in Mathematics.

[CR39] Russo J, Russo T, Kalogeropoulos P (2020). Exploring exponential growth in elementary school. Mathematics Teacher: Learning and Teaching PK.

[CR40] Schonger M, Sele D (2021). Intuition and exponential growth: Bias and the roles of parameterization and complexity. Mathematische Semesterberichte.

[CR41] Sezer HB, Namukasa IK (2021). Real-world problems through computational thinking tools and concepts: The case of coronavirus disease (COVID-19). Journal of Research in Innovative Teaching & Learning.

[CR42] Siller, H. S., & Greefrath, G. (2010). Mathematical modelling in class regarding to technology. *Proceedings of the Sixth Congress of the European Society for Research in Mathematics Education*, 2136–2145. www.inrp.fr/editions/cerme6

[CR43] Stillman, G., Kaiser, G., & Lampen, C. E. (Eds.). (2020). *Mathematical Modelling Education and Sense-making*. Springer International Publishing. 10.1007/978-3-030-37673-4

[CR44] Thiel-Schneider A (2018). Zum Begriff des exponentiellen Wachstums. Springer Fachmedien Wiesbaden.

[CR45] Vom Hofe R, Blum W (2016). “Grundvorstellungen” as a category of subject-matter didactics. Journal Für Mathematik-Didaktik.

[CR46] Vorhölter, K., Greefrath, G., Borromeo Ferri, R., Leiß, D., & Schukajlow, S. (2019). Mathematical modelling. In H. N. Jahnke & L. Hefendehl-Hebeker (Eds.), *Traditions in German-Speaking Mathematics Education Research* (pp. 91–114). Springer International Publishing. 10.1007/978-3-030-11069-7_4

[CR47] Vorhölter, K., & Schwarz, B. (2020). Fostering students’ construction of meaningfulness of mathematics with mathematical modelling problems. In G. Stillman, G. Kaiser, & C. E. Lampen (Eds.), *Mathematical Modelling Education and Sense-making* (pp. 323–333). Springer International Publishing. 10.1007/978-3-030-37673-4_28

[CR48] Wagenaar WA, Sagaria SD (1975). Misperception of exponential growth. Perception & Psychophysics.

